# Analysis of the compatibility of dental implant systems in fibula free flap reconstruction

**DOI:** 10.1186/1758-3284-4-37

**Published:** 2012-06-21

**Authors:** Ramin Carbiner, Waseem Jerjes, Kaveh Shakib, Peter V Giannoudis, Colin Hopper

**Affiliations:** 1 Head and Neck Centre, University College London Hospitals, London, UK; 2 Unit of Oral and Maxillofacial Surgery, UCL Eastman Dental Institute, London, UK; 3 Department of Surgery, UCL Medical School, London, UK; 4 Academic Department of Trauma and Orthopaedic Surgery, Leeds Teaching Hospitals NHS Trust, Leeds, UK; 5 Leeds Institute of Molecular Medicine, University of Leeds, Leeds, UK; 6 Department of Oral and Maxillofacial Surgery, Chase Farm Hospital, Enfield, UK

## Abstract

As a result of major ablative surgery, head and neck oncology patients can be left with significant defects in the orofacial region. The resultant defect raises the need for advanced reconstruction techniques. The reconstruction in this region is aimed at restoring function and facial contour. The use of vascularised free flaps has revolutionised the reconstruction in the head and neck. Advances in reconstruction techniques have resulted in continuous improvement of oral rehabilitation. For example, endosteal implants are being used to restore the masticatory function by the way of prosthetic replacement of the dentition. Implant rehabilitation usually leads to improved facial appearance, function, restoration of speech and mastication. Suitable dental implant placement’s site requires satisfactory width, height and quality of bone. Reconstruction of hard tissue defects therefore will need to be tailored to meet the needs for implant placement.

The aim of this feasibility study was to assess the compatibility of five standard commercially available dental implant systems (Biomet 3i, Nobel Biocare, Astra tech, Straumann and Ankylos) for placement into vascularised fibula graft during the reconstruction of oromandibular region.

Radiographs (2D) of the lower extremities from 142 patients in the archives of the Department of Radiology in University College London Hospitals (UCLH) were analysed in this study. These radiographs were from 61 females and 81 males. Additionally, 60 unsexed dry fibular bones, 30 right sided, acquired from the collection of the Department of Anatomy, University College London (UCL) were also measured to account for the 3D factor.

In the right fibula (dry bone), 90% of the samples measured had a width of 13.1 mm. While in the left fibula (dry bone), 90% of the samples measured had a width of 13.3 mm. Fibulas measured on radiographs had a width of 14.3 mm in 90% of the samples. The length ranges of the dental implants used in this study were: 7-13 mm (Biomet 3i), 10-13 mm (Nobel biocare), 8-13 mm (Astra Tech), 8-12 mm (Straumann ) and 8-11 mm (Ankylos).

This study reached a conclusion that the width of fibula is sufficient for placement of most frequently used dental implants for oral rehabilitation after mandibular reconstructive procedures.

## Introduction

As a result of major ablative surgery, head and neck oncology patients can be left with significant defects in the orofacial region. The resultant defect raises the need for advanced reconstruction techniques. The reconstruction in this region is aimed at restoring function and facial contour. The use of vascularised free flaps has revolutionised the reconstruction in the head and neck. In addition to restoring structure, these flaps have reduced the adverse effects of tumour surgery on the patient's oral function when compared to other reconstructive means. Advances in reconstruction techniques have resulted in continuous improvement of oral rehabilitation. For example, endosteal implants are being used to restore the masticatory function by the way of prosthetic replacement of the dentition. Implant rehabilitation usually leads to improved facial appearance, function, restoration of speech and mastication [1-16].

Several vascularised bone grafts have been used for the reconstruction of mandibular defects, such as the radius, metatarsus, thoracic rib, scapula, iliac crest, and fibula. Fibula free tissue transfer has demonstrated high reliability and adaptability for the reconstruction of those defects, due to its length (up to 25 cm), long vascular pedicle and ability to be osteotomised to provide a favorable facial contour [17]. The fibula flap can be used as an osteomuscular flap or osteomyocutaneous flap. The advantage of the latter is to provide a simultaneous reconstruction of intraoral defects (i.e. cheek, palate, floor of the mouth) and cutaneous defects in the same area. Moreover, the fibular bone (due to its appropriate thickness and bicortical nature), can act as a viable recipient site for implant placement and subsequent implant-supported prosthesis [4].

**Figure 1:**
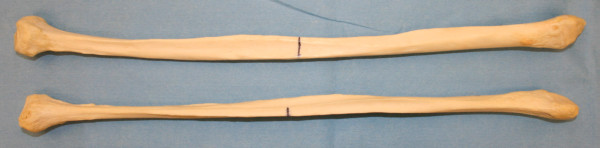
Left fibula bone (top) and a right fibula (bottom).

The transfer of the osseofasciocutaneus vascularised fibula free flap has become a routine procedure in the reconstruction of comprehensive oromaxillofacial defects. The fibula flap is being increasingly used for both mandibular and maxillary reconstruction. Modifications of the harvesting techniques have improved the reliability of the skin pedicle extending the application of this flap, although both the bone and the skin must share the same alignment [18]. The deficit(s) at the donor site is limited, and most patients are not troubled by them (i.e. pain, cosmesis…etc.). In contrast to the shape of the mandible, the fibula is a straight bone (Figure1). A prime advantage of the fibula is that up to 270 mm in length may be harvested, allowing reconstruction of any length of mandibular defect. Besides its length, major advantages of the fibula free flap include the trigonal diameter of the fibular bone, with the additional advantage of bicortical anatomy which usually allows the placement of dental implants and facilitate osseointegration [18].

**Figure 2:**
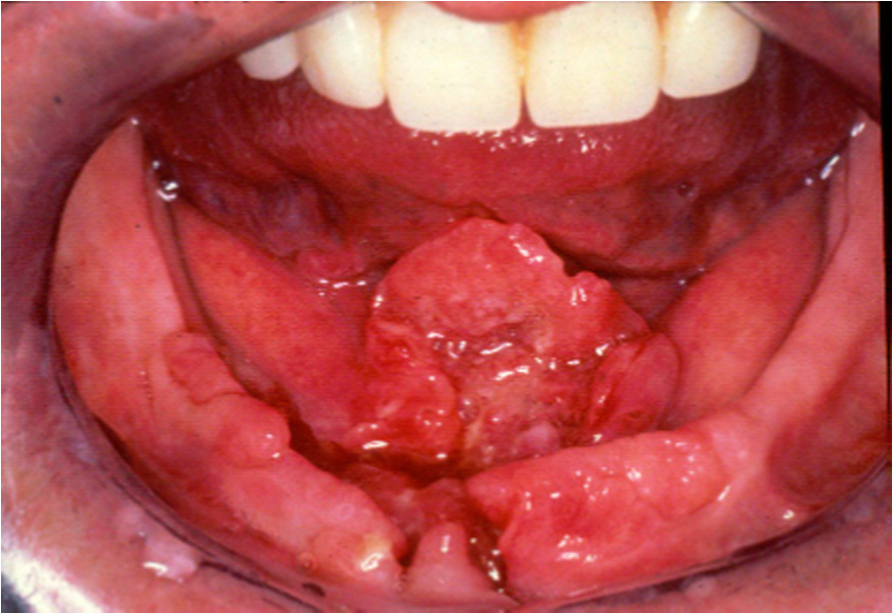
T4 SCC floor of mouth and alveolus.

**Figure 3:**
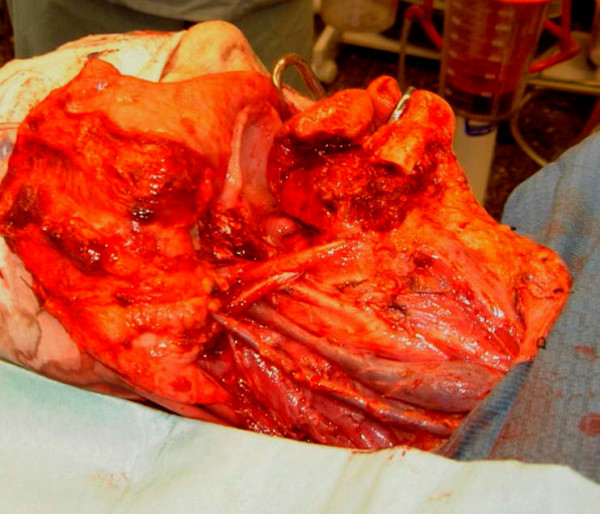
Right neck dissection levels I-IV.

**Figure 4:**
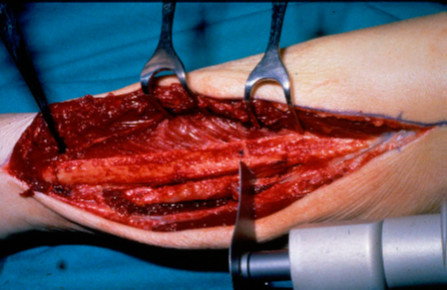
Dissected fibula ready for harvesting.

**Figure 5:**
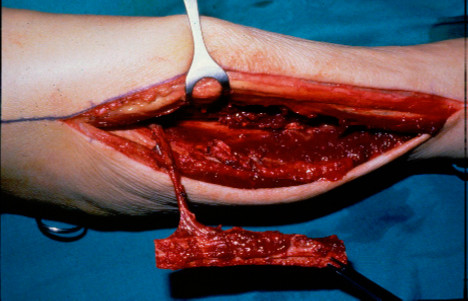
Harvested vascular fibular pedicle.

**Figure 6:**
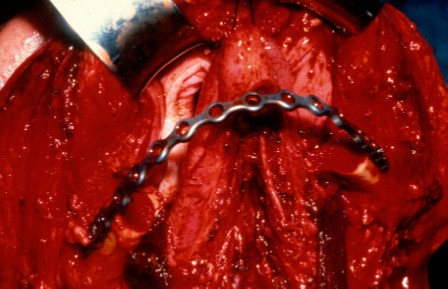
Reconstruction plate in place.

**Figure 7:**
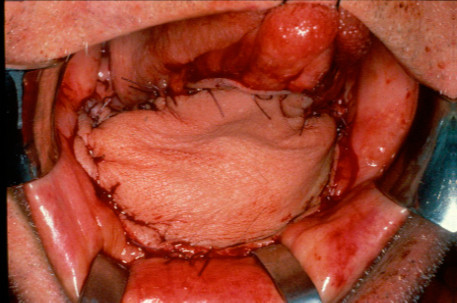
Anastomised fibular pedicle in placed.

**Figure 8:**
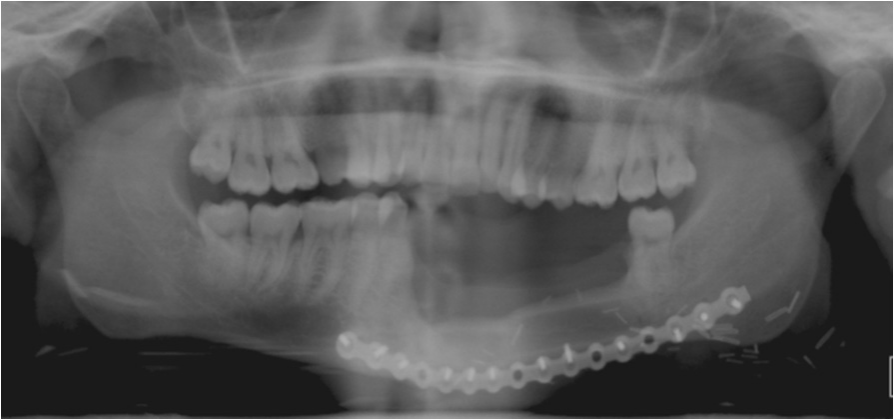
OPG shows a reconstructed mandible with vascularised fibula free flap after hemimandiblectomy as result of cancer surgery.

To restore mandibular anatomical continuity and configurations in the case of a mandibular defect, the fibula can be osteotomised at several places depending on the extent and location of the defect and used for reconstruction of the mandible [19]. An additional advantage is the option to harvest the fibula flap as an osseofasciocutaneous [2,20]. Although the skin paddle is suitable for oral reconstruction, it does not provide an appropriate peri-implant environment [17,18]. The resection of floor of mouth and alveolus carcinoma followed by reconstruction of the defect with free fibular flap is demonstrated in Figures2,3,4,5,6,7,8.

Optimal biomechanical and biochemical stimuli from the implant surface are of utmost importance for the bone healing process. The establishment and maintenance of a soft tissue seal around the transmucosal part of an implant (i.e. the abutment) is vital for implant treatment success [21,22].

There are more than 300 different dental implant systems in the market. The most frequently used in the reconstruction of the oromaxillofacial region include Biomet 3i, Nobel Biocare, Astra Tech, Straumann and Ankylos. Suitable dental implant placement’s site requires satisfactory width, height and quality of bone. Reconstruction of hard tissue defects therefore will need to be tailored to meet the needs for implant placement.

The aim of this feasibility study was to assess the compatibility of five standard commercially available dental implant systems (Biomet 3i, Nobel Biocare, Astra tech, Straumann and Ankylos) for placement into vascularised free fibulagraft during the reconstruction of oromandibular region.

## Materials & methods

The protocol of this study was approved by the UCL/UCLH Committee for Research Ethics Concerning Human Subjects.

**Figure 9:**
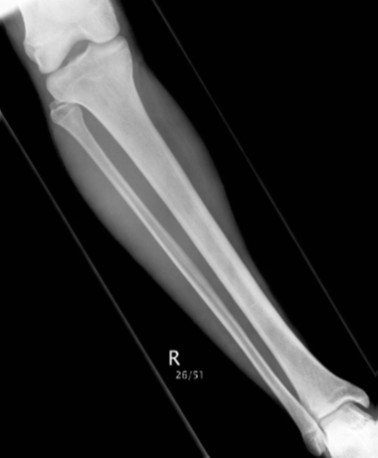
AP X-ray shows tibia and fibula.

Anterio-posterior (AP) digital radiographs (2D) of the lower extremities from 142 patients from the Department of Radiology at UCLH were included in this study. These radiographs were from 61 females and 81 males. Radiographs were accessed through the UCLH Picture Archiving and Communication System (PACS). Measurements were acquired by using the tools provided by the PACS system (i.e. electronic ruler). Exclusion criteria were patients who had any history of fractures, long-term use of steroids, poor quality X-rays of the tibia or fibula and degenerative bone disease. The inclusion criteria were patients above eighteen years of age (Figure9). The first step was to measure the fibular length on the radiograph; this was followed by registering the length midpoint and the width at the midpoint.

Sixty unsexed dry fibula bone, (30 right sided), were acquired from the specimens collection at the Department of Anatomy, UCL to account for the 3D factor. Fibula length and width measurement at the midpoint were acquired as in the previous cohort. Digital Verniar calliper was used to measure the width (Figure1).

Five of the most frequently used dental implant systems, in oral and maxillofacial surgery, were assessed for their feasibility in restoring function in reconstructed mandibles with vascularised fibula free flap. The measurements were obtained directly from the manufacturers and were not measured independently in this study.

### Statistical analysis

Data were analysed using the software SPSS for windows (Version 14.0 SPSS). When plotted, the means were normally distributed and a two Sample *t*-test has been used to look for the differences between the two groups (male and female) in AP X- ray measurements and differences in the right and left dry bones. When a P-value <0.001 (or 0.1 percent) was reported, it was assumed that, since both samples were normally distributed, the difference was significant.

## Results

Measurements taken from the 142 AP X-rays of the lower extremities showed the mean fibular length to be 399 mm (range male 339-446 mm and female 319- 436 mm), while the mean of the registered midpoint of the fibular length was 194 mm (range: male 170-223 mm and female 160-218 mm), the mean fibular width was 12 mm (range: male 9.4-17.4 mm and female 7.7-15.6 mm). On radiographs, male fibulas were found to be significantly longer and wider (P-value <0.001). Measurements of 60 unsexed dry fibular bones, showed the mean fibular length to be 360 mm (range 332-397 mm), while the mean midpoint was 180 mm (range: 166-198 mm), the mean fibular width at the registered midpoint was 10.5 mm (range: 6.67-14.2 mm); there was no significant difference in the length or width when comparing the right to the left fibulas.

The mean widths of fibulas on X-rays were significantly higher than dry bone taking into consideration the magnification factor (on the radiographs). Based on gender, it was found that the widths of male fibulas measured on radiographs are significantly higher. A 2 sample T- test has been performed with 95% confidence interval and showed a P-value of 0.772, 0.779 and 0.646 for the length, midpoint and width, respectively.

In the right fibula (dry bone), 90% of the samples measured had a width of 13.1 mm. While in the left fibula (dry bone), 90% of the samples measured had a width of 13.3 mm; fibulas measured on radiographs had a mean width of 14.3 mm in 90% of the samples.

With regards to the dental implants, Astra implants varied in length from 8 mm to 19 mm, while their diameter was from 3.5 mm to 5 mm. Ankylos implants also varied in length from 8 mm to 17 mm with a diameter of 3.5-7 mm. While Straumann implants length was 8-16 mm with a diameter 3.3-4.8 mm. Biomet 3i length can reach up to 20 mm with a diameter of 3.25-6 mm. With Noble Biocare, the implant length varies from 10- 15 mm while the width can reach up to 5 mm. When comparisons are made, it was obvious that the width of fibula is sufficient for placement of most frequently used dental implants for oral rehabilitation after mandibular reconstructive procedures.

## Discussion

Reconstruction of the oromandibular region following major resective surgery or severe comminuted facial fractures remains a complicated issue. The use of vascularised free tissue transfer with hard tissue, especially in vascularised fibula free flap, provides one of the best possibilities for full functional mandibular rehabilitation in combination with dental implants; whether such an approach should occur primarily in combination with the free flap reconstruction or in a later stage is debated; however many believe that it increases the survival of composite tissue flaps and enhances functional result [1-16,23].

Two aspects regarding the relevance of this feasibility study need exploring. First, is the width of bone component of the free fibula flap sufficient to receive the most frequently used dental implants? And second, is there any alternative technique that could be employed to improve the height of the hard tissue in the fibula free flap for better prosthetic rehabilitation which will ultimately improve the patient’s quality of life?

In our study, we looked at the compatibility of five different dental implant systems (namely: Biomet 3i, Nobel Biocare, Astra tech, Straumann and Ankylos) in fibula free flap reconstruction. A vertical bone height of 7-10 mm is frequently taken as the minimum bone height to be used for implant placement so for the purposes of this study, we established a minimum bone height criteria of 10 mm. Numerous studies have concluded that ≥10 mm represents a sufficient bone thickness in mandibular reconstruction for safe osteointegrated implant placement [23]. Also, according to previous studies, an implant diameter range of 3.75-4.8 mm is suitable for placement in reconstructed mandible with fibula free flap [18,24,25].

Fibular bones were measured on 142 AP X-rays taken from PACS radiographic database at UCLH and 60 unsexed dry bones at the Anatomy Department, UCL. The mean length, midpoint and width at the midpoint acquired from X-rays were 399 mm, 194 mm and 12 mm, respectively; and for dry bone, they were 360 mm, 180 mm and 10.5 mm, respectively, fibulas measured on radiographs had a width of 14.3 mm in 90% of the samples. Furthermore, 90% of the right fibula (dry bone) samples measured had a width of 13.1 mm. While in the left fibula (dry bone), 90% of the samples measured had a width of 13.3 mm.

The five implant systems sizes (length and diameter) were analysed and compared with the mean measurements of width of fibula on X-rays and dry bones. The outcome of the results revealed that any of the implant systems included in this study would allow the possibility of prosthetic rehabilitation. Also our results are in line with previous findings in the literature [23].

An important consideration in relation to fibula free flap and implantation is the irradiation delivered in most of the head and neck cancer patients in the immediate postoperative phase. After radiotherapy bone regeneration is depressed by 70.9% with a recovery of up to 28.9% in year one; the recommended time for attempting implantation is a minimum of 12 months after irradiation [17].

Reconstruction of mandibular defects following surgical ablation for tumours or after osteoradionecrosis with fibula free flaps has shown to be a reliable technique with good long-term prognosis. Implants placed in the reconstructed areas have been demonstrated to integrate normally with high success and survival rates when compared to those implants placed in native bone [4].

The creation of an adequate implant crown ratio with proper reconstruction of the alveolar processes, could improve implant position and angulations and consequently functional ability. The limited thickness of the fibular diaphysis prevents the use of implants longer than 10-12 mm. This limitation can result in unfavourable implant crown ratio. An unfavourable implant crown ratio produces bending moments, possible screw loosening, component fracture, or even implant fracture. Also, aesthetic problems and difficulties in obtaining adequate oral hygiene may be present [26]. To achieve better implant crown ratio after autogenous reconstruction of the mandible with fibula free flap, different techniques have been suggested including the use of double-barrelled reconstruction [27], distraction of the recipients site and additional free bone grafting, and corticocancellous iliac grafts.

In double-barrelled reconstruction, there is risk of blocking the blood supply to the graft which consequently causes flap failure. Distraction osteogenesis completely avoids problems like donor site morbidity, soft tissue limitations at the recipient site and unpredictable graft resorption [3,28-31].

A valid criticism of the method used in measuring the fibula is that 2D radiographs were used. This problem could have been avoided if 3 Dimensional Computed Tomography (3D CT) scans were used instead. However such scans were not available in significant numbers on the PACS radiographic database. The use of the dry skeleton was aimed in part to redress this shortcoming.

In addition, we must mention that so far there is no particular dental implant system specifically designed for the use in reconstructed mandible with fibula free flap. However all five dental implant systems included in this study were shown to be suitable for the placement in reconstructed mandible with fibula free flap.

To sum up, oromandibular reconstruction is a complex procedure with many available options. Mandibular defects could be better reconstructed with fibula free flap, with recognised improvements in the patient’s prognosis (i.e. facial contour and function). We can conclude from this feasibility study that the width of fibula is sufficient for placement of the most frequently used dental implants for oral rehabilitation after mandibular reconstructive procedures. However for optimal prosthetic rehabilitation, distraction osteogenesis is preferred. Furthermore, double-barreled reconstruction is one of the treatment options which give an instant increase in height of mandible for more precise placement of implant and possibility for an optimal oral rehabilitation.

## Competing interests

The authors declare that they have no competing interests.

## Authors’ contributions

All authors contributed to conception and design, carried out the literature research, manuscript preparation and manuscript review. All authors read and approved the final manuscript.
